# Is there a Role for Antibiotic Prophylaxis Prior to Dental Procedures in Patients with Total Joint Arthroplasty? A Systematic Review of the Literature

**DOI:** 10.7150/jbji.40096

**Published:** 2020-01-01

**Authors:** Pablo Ariel Slullitel, José Ignacio Oñativia, Nicolás Santiago Piuzzi, Carlos Higuera-Rueda, Javad Parvizi, Martín Alejandro Buttaro

**Affiliations:** 1 Hip Surgery Unit, Institute of Orthopaedics “Carlos E. Ottolenghi”, Italian Hospital of Buenos Aires, Buenos Aires, Argentina.; 2 Division of Orthopaedic Surgery, The Ottawa Hospital, Ottawa, Ontario, Canada.; 3 Adult Reconstructive Surgery, Division of Orthopaedic Surgery, Cleveland Clinic, Ohio, United States.; 4 Rothman Orthopaedic Institute at Thomas Jefferson University, Philadelphia, PA; United States.

**Keywords:** periprosthetic joint infection, haematogenous infection, total joint arthroplasty, dental procedure, antibiotic prophylaxis

## Abstract

**Background**: The indication of prophylactic antibiotics prior to dental procedures for non-infected causes in order to reduce the risk of haematogenous periprosthetic joint infection (PJI) remains as controversial. We performed a systematic review of the literature assessing the relationship between PJI and invasive dental procedures and whether there is evidence to support the use of antibiotic prophylaxis.

**Methods**: This review was conducted in accordance with the 2009 Preferred Reporting Items for Systematic Review and Meta-Analysis (PRISMA) statement. MEDLINE, EMBASE and the Cochrane Database of Systematic Reviews were searched for studies focusing on dental procedures after TJA, reporting on PJI as an outcome. The methodological quality was assessed with the Newcastle-Ottawa quality assessment scale for case-control and cohort studies and by the tool proposed by Murad et al. for observational studies.

**Results**: Our systematic literature review yielded 90 individual studies, of which 9 met the inclusion criteria. The overall infection rate ranged from 0.26% to 2.12%. Of these, cases associated with a dental procedure ranged from 0% to 15.9%. Five of the studies described cases in which antibiotic prophylaxis was administered; however, no clear algorithm regarding type and dosage of antibiotic was mentioned. When assessing the methodological quality of the evidence, all studies had an overall low to moderate quality.

**Conclusion**: The current systematic review, mostly composed of low-quality studies, suggests that there is no direct evidence to indicate prophylactic antibiotics prior to dental procedures in patients with TJA. In line with the current guidelines, no prophylaxis should be used on interventions for non-infected causes, except for occasional unusual situations, which can then be judged individually.

## Introduction

Periprosthetic joint infection (PJI) constitutes a severe complication after total joint arthroplasty (TJA), being the leading cause of revision after primary total knee replacement (TKR) and the third following primary total hip replacement (THR). Currently, PJI has a prevalence of 1%-2% in TKR^1^ and of 0.3%-2.9% in THR cases.^2^

Haematogenous PJIs can occur as a result of spread of infective organisms from a distant anatomic location, such as the heart, the lungs, skin, urinary tract and the oral cavity to a prosthetic joint.^3^ Reports indicate that up to one-third of all PJI may be haematogenous in nature.^4^ ^5^ The evidence behind the indication of antibiotic prophylaxis in an effort to minimize the spread of oral pathogens to the prosthetic joint is scant and administration of antibiotic prophylaxis to TJA patients undergoing a dental procedure remains a point of controversy.^6^

Numerous organizations such as the American Association of Orthopaedic Surgeons (AAOS), the American Dental Association (ADA), and the International Consensus Meeting (ICM) on Orthopaedic Infections have engaged in developing guidelines or recommendations related to this practice. The guidelines issued by the ADA in 2014 explicitly mention “that the current evidence shows no association between dental procedures and PJI nor a protective benefit of antibiotic prophylaxis before dental procedures. Therefore, for patients with prosthetic joints, antibiotics should not be prescribed prior to dental procedures to prevent prosthetic joint infection”.^7^ The recommendations from the AAOS and ICM, on the other hand, are not so conclusive leading to incongruences in recommendations and variabilities in practice.^8^

Since there is still lack of compelling evidence resulting in an unclear balance between benefits and potential harms in administration of prophylactic antibiotics prior to dental procedures performed to treat a non-infectious pathology at the oral cavity of patients with TJA, we decided to perform a systematic review of the subject.

## Materials and Methods

This review was conducted in accordance with the 2009 Preferred Reporting Items for Systematic Review and Meta-Analysis (PRISMA) statement.^9^ We also followed the MOOSE Guidelines for Meta- Analyses and Systematic Reviews of Observational Studies.^10^

### Data sources and searches

MEDLINE, EMBASE, and the Cochrane Database of Systematic Reviews and the Cochrane Central Register of Controlled Trials were searched for studies enrolling patients older than 18 years old, focusing on dental procedures after TJA, reporting on the correlation between PJI and antibiotic prophylaxis as an outcome. The search strategy was as follows: (((((arthroplasty[MeSH Terms]) AND "antibiotic prophylaxis") AND "dental") AND "humans")) AND ("1980/01/01"[Date - Publication]: "2017/12/31"[Date - Publication]). The search was performed in February 2018, limited to humans and restricted to publications after 1980. Additionally, the reference lists of included studies were screened to minimise the risk of missing relevant articles.

### Study selection

Three investigators independently screened title and abstracts of all the identified references. Then, full-text articles of studies that satisfied the selection criteria were retrieved and assessed by pairs of independent authors to confirm eligibility. Disagreements were solved by consensus. Therapeutic or prognostic studies, published in English or Spanish language, investigating the incidence of PJI after dental procedures with special interest in antibiotic prophylaxis were included. Letters to the editor or editorials, reviews, guidelines, commentaries, case reports and articles based on cadaveric or animal subjects, were excluded.

### Data extraction and quality assessment

Level of evidence of the included studies was assigned using the classification suggested by Wright et al.^11^ Patient demographics, time to dental procedure, dosage of antibiotic prophylaxis and outcome assessment in terms of haematogenous PJI diagnosis were recorded for each study into a custom data collection form.^12^

To assess the methodological quality of the included case-series studies, we utilized a tool developed by Murad et al. that includes 8 questions summarized in 4 global domains, including selection, ascertainment, causality and reporting.^13^ Affirmative response to each question allows building up a score rated from 0 to 8 points; the higher the score, the greater the likelihood of the study to avoid chance, biases and confounding factors. Two independent authors rated the quality of each published study. Disagreement was solved by consensus or consultation with the senior reviewer. In turn, the Newcastle-Ottawa scale for nonrandomized studies was used when assessing the methodological quality of cohort studies; this tool evaluates three categories that are selection (with a maximum of 4 stars score), comparability (2 stars maximum) and exposure (3 stars maximum).^14^

### Data synthesis

Due to the identification of only 1 controlled study, and according to the methodological recommendation against performing a meta-analysis of observational studies without a control group,^15^ information regarding outcomes reported by each study was presented in tables for comparison without a statistical assessment.

## Results

**Figure 1:**
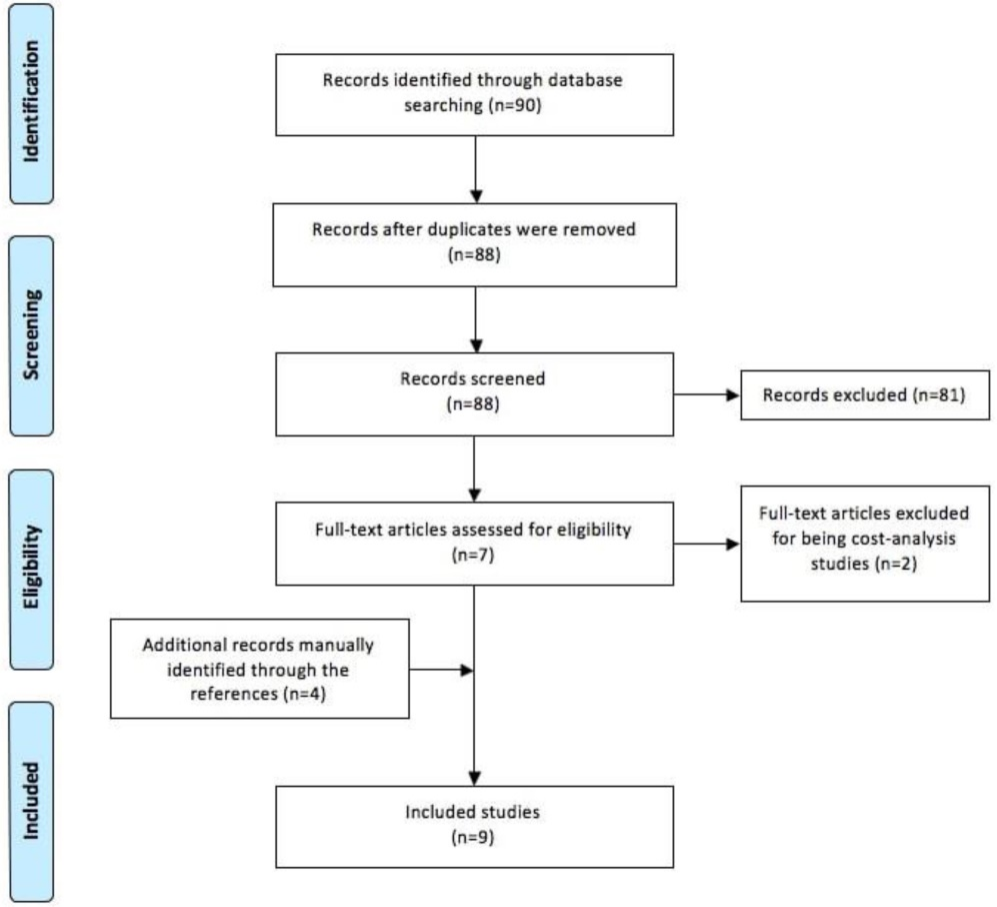
Flow diagram showing the systematic review process used in this study.

Our systematic literature review yielded 90 individual studies, of which only 9 met the inclusion criteria.^16^,^17^,^18^,^19^,^20^,^21^,^22^,^23^,^24^ Five studies were selected from the index search after initial exclusion per title and abstract; the remaining 4 were manually added from the references of the former ones (Figure 1). Two studies resulting from the index search initially selected were later excluded for being cost-analysis.

According to the Oxford Centre for Evidence-Based Medicine 2011 Levels of Evidence,^25^ 6 studies corresponded to level IV, 2 studies to level III and 1 study to level I. The methodological quality tool evidenced an overall low quality of the included case series, scoring a mean of 5,16 points (range 4 to 7) (Table 1). The methodological quality of the cohort studies is presented on Table 2, with the study Berbari et al.^22^ having the highest score when compared to the ones by Skaar et al.^23^ and Kao et al.^24^. In summary, the methodological assessment showed great heterogeneity in terms of study design and outcome assessment. Three of the studies were prospective in nature and the remaining were retrospecttive, 6 of them being case series, two case-control and only one retrospective cohort study. All were conducted between 1980 and 2016; 7 included patients treated at a single institution, while 2 included data collected from research databases (Taiwan National Registry^24^ and Medicare Registry^23^).

Except for Berbari et al.,^22^ none of the studies aimed specifically at studying if a prophylaxis before dental interventions would prevent from haematogenous PJI of oral origin, but rather investigated the epidemiology of haematogenous PJI per se. After obtaining each patient's dental record, Berbari et al. compared 339 PJI cases with 339 TJA controls without PJI who were hospitalized either for an arthroplasty located at a different site from the index arthroplasty, for an aseptic revision of the index arthroplasty, or for other orthopaedic procedure.^22^ Based on the complexity of the dental procedure, they divided the cohort into low- and high-risk groups and performed a dental propensity score depending on whether the patients received antibiotic prophylaxis or not, had dental visits or not and whether they were edentulous or not.^22^

### Demographics

Table 3 summarizes the demographic characteristics of the included studies. Two articles did not describe patients' age.^16^ ^24^ Mean time to dental procedure from index arthroplasty, reported by 5 articles, ranged from 15 to 180 months.^16^ ^17^ ^18^ ^20^ ^22^ None of the studies reported the follow-up period after infection diagnosis.

### Overall periprosthetic joint infection outcome

Eight studies reported on overall incidence of PJI, except for the study by Berbari et al.^22^ who centred their analysis purely on infected joint replacements without mentioning an initial number of overall arthroplasty cases (Table 4). Overall, the mean infection rate ranged from 0.26% to 2.12%, with the study by Kao et al.^24^ exhibiting the lowest frequency and study by Skaar et al.^23^ the highest rate of infection.

### Periprosthetic joint infection associated with a dental procedure

All of the studies focused on the diagnosis of PJI occurring after a dental procedure. Of the total infections, those associated with a dental procedure ranged from 0% to 15.9% (Table 4), based on a timely association between PJI and prior dental intervention. However, the source of infection was not certain in any of the studies. In fact, only 4 articles mentioned the infective organism.^16^ ^18^ ^20^ ^24^

Jacobsen and Murray reported that the only PJI they found was linked to the endodontic and antibiotic (unspecified) treatment of a periapical abscess.^16^ Uçkay et al. explained that the 3 dental procedures associated with a PJI were performed in cases with an already established dental abscess.^21^ Except for LaPorte et al.,^20^ none of the remaining studies made it clear whether the dental procedures had been performed on already established dental infections. Finally, Berbari et al. distinguished amongst low-risk (restorative dentistry, dental filling, endodontic treatment, and fluoride treatment) and high-risk dental procedures (dental hygiene, mouth surgery, periodontal treatment, dental extraction, and therapy for dental abscess).^22^ Nonetheless, the authors did not express in numbers how the diagnoses were distributed in each cohort.

The most common isolated bacteria, in decreasing order of prevalence, were as follows: *Streptococcus viridans*, *Peptostreptococcus*, *β-haemolytic Streptococcus*. Mean time to the diagnosis of PJI after the dental procedure was not clearly reported in these studies either.

### Antibiotic prophylaxis

All but one^19^ study mentioned whether an indication of antibiotic prophylaxis prior to dental procedure was in place for the patients with TJA in their cohort. Five of the studies mentioned that antibiotic prophylaxis was indicated prior to dental procedures,^16^ ^18^ ^22^ ^23^ ^24^, but only 2 studies described the dose and time of administration of antibiotics.^16^ ^23^ None of the studies disclosed the algorithm for antibiotic prophylaxis prior to dental procedures in their cohort (Table 5).

### Risk factors for PJI following dental procedures

The study by Jacobsen and Murray was unable to show a direct association between infection in hip prosthesis and dental treatment for an infectious cause located at the oral cavity.^16^ Similarly, Skaar et al. were unable to support the hypothesis that dental procedures can lead to PJI.^23^ After performing a multivariate analysis, Kao et al. found that dental procedures were not linked to the occurrence of PJI.^24^ As a sensitivity test, their stratified analyses consistently revealed among various subgroups that there was no association between dental procedure and PJI.

Waldman et al. suggested that extensive, but not routine low-risk, dental procedures may be associated with infections.^18^ Similarly, LaPorte et al. described that infections were more frequently associated with more invasive dental procedures such as multiple tooth extractions, root-canal operations and periodontal procedures.^20^ Conversely, Berbari et al. found that neither low-risk nor high-risk dental procedures were associated with PJI. The authors of the same study stated that patients with more than 1 dental hygiene visit per year were 30% less likely to develop prosthetic hip or knee infection, although this ascertainment was not statistically significant.^22^ Berbari et al.^22^ and Skaar et al.^23^ emphasized that dental procedures done within the first 2 years after the joint replacement were not associated with an increased risk of PJI.

Cook et al. highlighted the importance of counselling patients, especially those with known risk factors, that they may develop an infection in their replaced joint at any time.^19^ The multivariate logistic regression analysis done by Uçkay et al. revealed that body mass index, duration of surgery >180 minutes and revision arthroplasty were significantly associated with infections of any origin, including both primary surgical site infections and haematogenous infections.^21^ In this study, patients sustaining a haematogenous infection did not substantially differ from those with early acute infections, except that they were older (77 years vs. 68 years, respectively) and had a trend towards a higher prevalence for diabetes mellitus (29% vs. 11%, respectively). The study by Ainscow et al. did not report an analysis of factors related to PJI.^17^

## Discussion

The practice of administering antibiotic prophylaxis before dental procedures in patients with a prosthetic joint is not based on any evidence, and there are conflicting recommendations regarding it. The objective of this systematic review was to evaluate all published data, including publications that have emerged since the latest guidelines by the American Dental Association (ADA), related to administration of antibiotics to patients with TJA undergoing dental procedures done for non-infected causes. Our study reveals that there is no direct evidence that antibiotic prophylaxis has an impact on the incidence of haematogenous PJI after dental procedures.

The current study has several limitations that need to be born in mind when interpreting the findings. First, and foremost, available studies on this subject are relatively low quality, with many being retrospective in nature. Therefore, our review assumed all of the biases inherent to each of the included studies' design. Second, confirmation of a dental procedure as the potential source of PJI would require that the pathogen causing PJI would be an oral organism cultured from the mouth, blood, and the infected joint simultaneously.^26^ None of the studies, however, provided such evidence. Thus, the reported percentage of infections associated with dental procedures should be considered as best-case estimates. Third, the missing critical data (i.e., lack of pathogen isolation from both surgical site and oral cavity) from these studies and the retrospective nature of many included studies did not allow us to perform appropriate multivariate analysis to examine the association between dental procedures and subsequent PJI. Fourth, it should be highlighted that most of the studies were not aimed at analysing the effectiveness of antibiotic prophylaxis. Except for Berbari et al.,^22^ all studies focused their analysis on the epidemiology of haematogenous PJI, and were therefore designed without a precise methodology in order to address the aim of our systematic review.

The studies included reported a relatively low rate of PJI in their cohorts and the source of infection could not be confirmed in any case. In other words, none of the studies could certify that dental procedures were an independent risk factor for haematogenous PJI. This issue might be related to most of the included studies being underpowered, increasing the probability of making a type II error.^27^ The proportion of PJI cases being haematogenous in nature varies between 20%-35%.^28^ However, Rakow et al. suggested that these figures might be underestimated since most reports fail to identify the route or source of infection.^3^ Maderazo et al. noticed that skin and soft-tissue infections were the leading primary focus of haematogenous PJI, with dental origin being responsible for only 15% of haematogenous PJI in their cohort.^29^ These figures do not seem to be changing over time, since a more recent article described a similar prevalence (11%) of dental haematogenous PJI.^3^

The distinction between haematogenous PJI derived from an infection in the mouth and from a dental procedure in a not yet infected area needs to be addressed. We found only 2 studies reporting on PJI derived from actual dental infections;^16^ ^21^ whereas the rest of the studies focused their analysis purely on dental procedures, one of them characterising the treatment of dental abscess, among other treatments, as a high-risk procedure.^22^ The latest report of the ADA made it clear that there is no evidence to support an association between dental procedures and the risk of experiencing PJI, and therefore no clear indication for antibiotic prophylaxis in these cases.^30^ On the other hand, the treatment of a dental abscess usually consists of draining, removal of the infectious tooth source and antibiotic support.^31^ Therefore, we believe that the recommendations of dental prophylaxis in TJA patients should not be based on literature that includes cases with established dental infections, which are a well-documented stronger source of haematogenous seeding,^32^ since this could be a potential confounding factor. On this basis, a panel composed of ADA and AAOS members developed an appropriate-use criteria (AUC) as a tool to define narrow clinical scenarios that would potentially benefit from antibiotic indication (i.e. immune-compromised patients with a dental abscess involving manipulation of the periapical or gingival tissue).^30^ ^33^ Nonetheless, despite this panel being composed of experts, this tool is not completely evidence-based.

Additionally, we consider that time from the dental procedure done for non-infected causes to the development of a PJI are a variable that may lead to misinterpretation of the results. None of the included studies focused on the mean time to PJI after the dental procedure was performed, as haematogenous infection is believed to occur more frequently in the early years after index arthroplasty. Prior animal and clinical studies demonstrated a higher likelihood of haematogenous infections during the first 2 years following an arthroplasty, that decreased substantially in the following years.^34^ ^35^ The speculative explanation for the possible higher likelihood of haematogenous spread of bacteria from dental procedures to a prosthetic joint in the early period after arthroplasty is based on the fact that active local inflammation and the osseointegration activity around uncemented components may lead to a higher blood flow to the prosthetic joint and the potential for seeding of organisms onto the implant surface.^6^ ^36^ Nevertheless, the current literature still does not show any clear relationship between time of implantation and haematogenous PJI. 36 37 In fact, two studies included in this systematic review did examine the latter issue and stated that dental procedures within the first 2 years after index arthroplasty were not associated with an increased risk of PJI.^22^ ^23^ We believe that time is a potential confounding factor that was not clearly addressed in the included articles. Since no culture samples from the oral cavity and bloodstream were available to confirm the origin of the PJI, the association between dental procedures and PJI was based only on time interval, which lacks scientific background.

The issue of administering antibiotics to TJA patients before procedures in the oral cavity without an active infection is important on many accounts. The first issue relates to antibiotic stewardship. Liberal use of antibiotics can lead to emergence of resistant organisms with grave consequences for the society.^38^ Based on the United States Census Bureau and the Nationwide Inpatient Sample, by 2030, the demand for primary THR and for TKR is estimated to grow by 174% to 572,000, and by 673% to 572,00, respectively.^2^ Thus, not an insignificant amount of antibiotic needs to be administered to TJA patients assuming each patient visits the dentist once or twice a year.^39^ There is a significant cost associated with administration of antibiotic to every patient with TJA undergoing dental procedure.^40^ Even if one were to assume that antibiotic prophylaxis during dental procedures is likely to reduce PJI, the cost-effectiveness of this practice has not been clearly demonstrated. Although individual costs of pre-dental procedure prophylaxis are low, overall costs for the American healthcare system are speculated to be high.^41^ ^42^ In fact, Lockhart et al. estimated an annual cost of between $19,880,279 and $143,685,823 in a population of 20 million people.^41^ Several studies compared the cost-effectiveness of prophylaxis with penicillin versus no prophylaxis, concluding that the low prevalence of haematogenous PJI makes it unnecessary to implement antibiotic coverage for every patient with TJA in place.^35^ ^43^ Following a Markov cost-effectiveness decision model, Skaar et al. recently found that the no-prophylaxis strategy is cost-effective for TKR patients without medical conditions, having the lowest average lifetime costs ($17,119) and quality-adjusted life years (11.2151).^44^ Finally, administration of antibiotics to individuals is not without its problem and can result in adverse events such as *Clostridium difficile* infection and other serious issues, such as the risk for anaphylaxis and the development of drug-resistant bacteria.^7^

In the absence of evidence supporting the role of antibiotic prophylaxis for every TJA patient undergoing dental procedure for a non-infectious cause, the recommendation of the International Consensus Meeting (ICM) is that prophylaxis should be reserved to patients with extensive comorbidities in whom the probability of developing PJI is higher or those with complex reconstructive procedure in whom development of PJI may have more dire consequences.^45^

## Conclusion

Haematogenous PJIs may develop following dental procedures, independently of the time of implantation of the prosthetic components. The current evidence, mostly composed of low-quality studies in terms of design, suggests that there is no proof to indicate antibiotic prophylaxis in patients with TJA undergoing dental procedures. In line with the current guidelines, no prophylaxis should be used on interventions for non-infected causes, except for occasional unusual situations, which can then be judged individually. High-quality evidence is necessary to further analyse the true efficacy of this medical practice and until then, this will likely remain a controversial aspect of our practice. Furthermore, since the estimated proportion of PJI cases attributed to dental procedures is trivial, the development of a randomized controlled trial will remain unfeasible considering the number of cases that should be included in order to reach a power analysis of 80%.^46^

## Figures and Tables

**Table 1:** Analysis of the methodological quality of case-series

Domain	Leading explanatory question	Jacobsen and Murray 1980	Ainscow et al. 1984	Waldman et al. 1997	Cook et al. 2007	La Porte et al. 2008	Uçkay et al. 2009
Selection	1. Do the patients represent the whole experiences of the center or is the selection method unclear (other patient with similar presentation may not have been reported)?	Yes	Yes	Yes	Yes	Yes	Yes
Ascertain-ment	2. Was the exposure adequately ascertained?	No	Yes	Yes	No	Yes	Yes
	3. Was the outcome adequately ascertained?	Yes	Yes	Yes	Yes	Yes	Yes
Causality	4. Were other causes that may explain the observation ruled out?	No	No	Yes	No	No	Yes
	5. Was there a challenge/rechallenge phenomenon?	Yes	Yes	Yes	Yes	Yes	Yes
	6. Was there a dose-response effect?	No	No	No	No	No	No
	7. Was follow-up long enough for outcome to occur?	N/A	Yes	Yes	Yes	N/A	Yes
Reporting	8. Are the cases described with sufficient details to allow other investigators to replicate the research or to allow practitioners make inferences related to their own practice?	Yes	No	Yes	No	Yes	No
Overall Score		4	5	7	4	5	6

**Table 2:** Analysis of the methodological quality of case-control and cohort studies

	Newcastle-Ottawa Quality Assessment Scale		
	Selection	Comparability	Exposure/Outcome
**Berbari et al. 2010**	✪✪✪✪	✪✪	✪✪
**Skaar et al. 2011**	✪✪	✪✪	✪✪
**Kao et al. 2016**	✪✪✪	✪	✪✪

**Table 3:** Demographic characteristics of the included studies.

Author	Study Design	Number of cases/Joint involved	Age (years)	Mean time to dental procedure (months)
Jacobsen and Murray 1980	Retrospective case series	1855 THAs (n=1729 received prophylaxis)	-	34 (Range, 17-48)
Ainscow et al. 1984	Prospective case series	885 THAs and 115 TKRs (n=128 had dental procedures)	70 (Range, 49-85)	72 (Range, 36-180)
Waldman et al. 1997	Retrospective case series	3564 TKRs	65 (Range, 56-76)	72 (Range, 26-95)
Cook et al. 2007	Restrospective case series	3013 TKAs	67 (range 40 - 79)	-
La Porte et al. 2008	Retrospective case series	2973 THAs	71 (Range, 63-78)	26 (Range, 15-39)
Uçkay et al. 2009	Prospective case series	4002 THAs and 2099 TKRs	69.9 (SD ±11.4)	-
Berbari et al. 2010	Prospective case-control	339 infected TJAs9 infected TJAs339 infected TJAs339 infected TJAs339 infected TJAs	Median 69.5 (Range, 25.7-91.2)	Low-risk procedure (<=12 months n=82; 12-24 months n=18); High-risk procedure (<12 months n=115; 12-24 months n=13)
Skaar et al. 2011	Retrospective case-control	468 THAs, 501 TKRs and 31 replacements of another joint	-	-
Kao et al. 2016	Cohort Study (with sub-cohorts)	255568 TJAs (n=61917 underwent dental procedures and 193651 did not undergo dental procedures)	50.17± 18.46	-

THA: Total hip arthroplasty; TKR: Total knee replacement; TJA: Total joint arthroplasty.

**Table 4:** Description of the included studies' infection outcome.

Author	Overall infection outcome in the whole cohort	Fraction of PJI related to dental work	Infecting organism associated with post-dental procedure infection
Jacobsen and Murray 1980	n=33/1855 (1.77%)	n=1/33 (3.03%)	*Staphylococcus aureus* (n=1)
Ainscow et al. 1984	n=22/1112 (1.97%)	n=0/22 (0%)	-
Waldman et al. 1997	n=74/3490 (2.12%)	n=9/74 (12%)	*Streptococcus viridans* (n=3), *Peptostreptococcus* (n=3), *Staphylococcus aureus* (n=1), *Serratia marcescens* (n=1), Mixed flora (n=1)
Cook et al. 2007	n=15/3013 (0.49%)	n=1/15 (6.6%)	-
La Porte et al. 2008	n=52/2973 (1.74%)	n=3/52 (5.76%)	*Streptococcus viridans* (n=2), *Peptostreptococcus* (n=1)
Uçkay et al. 2009	n=71/6101 (1.16%)	n=3/71 (4.22%)	*Streptococcus oralis* (n=1), *Streptococcus milleri* (n=1), *Staphylococcus aureus* (n=1)
Berbari et al. 2010	N/A (100% of cases had a PJI)	n=35/339 (10.3%)	*Beta-hemolytic streptococci* (n=13), *Peptostreptococcus* (n=5), *Actinomyces* (n=1), *Streptococcus viridans* (n=11), *Streptococcus-like organisms* (n=2), *Abiotrophia/Granulicatella* (n=2), *Gemella* (n=1).
Skaar et al. 2011	n=18/1000 (1.8%)	n=4/42 (9.52%)	-
Kao et al. 2016	n=676/255,568 (0.26%)	n=328/57,066 (0.57%) in cases with dental procedures (Vs. n=348/57,066 [0.61%] in the non-dental cohort)	-

PJI: Periprosthetic joint infection; Vs.: Versus; PJI: Periprosthetic joint infection.

**Table 5:** Indication of antibiotic prophylaxis and dosage characteristics.

Author	Prophylactic antibiotic before dental procedure (yes/no)	Antibiotic (if used)	Dosage of prophylactic antibiotic
Jacobsen and Murray 1980	Yes	Cephalotin + Erythromycin	-
Ainscow et al. 1984	No	-	-
Waldman et al. 1997	No (n=8), Yes (n=1)	Penicillin (in 1 case)	First generation cephalosporin given 1 hour preoperatively and 8 hours postoperatively
Cook et al. 2007	N/R	-	-
La Porte et al. 2008	No	-	-
Uçkay et al. 2009	No	-	-
Berbari et al. 2010	Low risk procedure (n=59/41); High risk procedure (n=95/33)	-	-
Skaar et al. 2011	Yes	-	-
Kao et al. 2016	Each cohort was divided in equally-distributed sub-cohorts with and without antibiotic prophylaxis	First- or second-generation cephalosporin, penicillin (e.g., oxacillin, ampicillin, and amoxicillin), or clindamycin	Within 1 week preceding the dental procedure
